# A New Cause of Obesity Syndrome Associated with a Mutation in the Carboxypeptidase Gene Detected in Three Siblings with Obesity, Intellectual Disability and Hypogonadotropic Hypogonadism

**DOI:** 10.4274/jcrpe.galenos.2020.2020.0101

**Published:** 2021-02-26

**Authors:** Asude Durmaz, Ayça Aykut, Tahir Atik, Samim Özen, Durdugül Ayyıldız Emecen, Aysun Ata, Esra Işık, Damla Gökşen, Özgür Çoğulu, Ferda Özkınay

**Affiliations:** 1 Ege University Faculty of Medicine, Department of Medical Genetics, İzmir, Turkey; 2 Ege University Faculty of Medicine, Department of Pediatrics, Subdivision of Pediatric Genetics, İzmir, Turkey; 3 Ege University Faculty of Medicine, Department of Pediatrics, Subdivision of Pediatric Endocrinology, İzmir, Turkey

**Keywords:** Obesity, hypogonadotropic hypogonadism, carboxypeptidase, carboxypeptidase E

## Abstract

**Objective:**

Carboxypeptidase E (CPE) plays a critical role in the biosynthesis of peptide hormones and neuropeptides in the endocrine system and central nervous system. *CPE* knockout mice models exhibit disorders such as diabetes, hyperproinsulinaemia, low bone mineral density and neurodevelopmental disorders. Only one patient is described with morbid obesity, intellectual disability, abnormal glucose homeostasis and hypogonadotropic hypogonadism, which was associated with a homozygous frameshift deletion in *CPE*.

**Methods:**

Herein are described three siblings with obesity, intellectual disability and hypogonadotropic hypogonadism. Whole exome sequencing (WES) was performed in the index case. Candidate variants were prioritised and segregation of the variant, consistent with the phenotype of the index case, was assessed by Sanger sequencing in affected siblings and parents.

**Results:**

WES analysis revealed a homozygous nonsense c.405C>A (p.Y135*) mutation in *CPE*. Validation and segregation analysis confirmed the homozygous mutation in the index case and his affected siblings. The parents were phenotypically normal heterozygous mutation carriers.

**Conclusion:**

This study provides additional evidence of the association between a homozygous nonsense mutation in *CPE* and a clinical phenotype consisting of obesity, intellectual disability and hypogonadotropic hypogonadism, which may be considered as a new monogenic obesity syndrome.

**What is already known on this topic?**

Various genetic factors play a role in childhood obesity. Mutations in the carboxypeptidase E (*CPE*) gene lead to the inactivation of the *CPE* enzyme, resulting in obesity, age-dependent infertility, hyperglycaemia, disorders of bone metabolism, inflammatory bowel disease and neurological abnormalities in mouse models.

**What this study adds?**

This study represents a potential disease-causing mutation in *CPE* which is not linked to a specific Mendelian syndrome in the Online Mendelian Inheritance in Man database. Together with only one previously reported case, this study confirms *CPE* as a novel form of Mendelian obesity syndrome.

## Introduction

Obesity and intellectual disability are found in many genetic disorders, ranging from monogenic disorders to microdeletion syndromes. This suggests that multiple genes are involved or share common pathways in obesity and intellectual disability. Carboxypeptidase E (CPE), which is enriched in mature secretory vesicles, was the first identified secretory pathway carboxypeptidase (1). It plays a major role in the biosynthesis of peptide hormones and neuropeptides in endocrine and neuroendocrine cells (2). CPE also serves as a regulated, secretory pathway-sorting receptor for many peptides, including proinsulin, proenkephalin, proopiomelanocortin (POMC) and brain derived neurotrophic factor (NF) (BDNF) (3). Appetite–controlling neuropeptides, such as POMC must be cleaved into several biologically active peptides, including α–melanocyte stimulating hormone and β–endorphin to inhibit food intake. This cleavage is mediated by prohormone convertases such as PC1, PC2, CPE, and peptidyl α–amidating monooxygenase suggesting that inactivated PC1 and CPE play a role in obesity (4). The *CPE* gene is not linked to a specific human syndrome. However, some mouse model studies indicate its role in pathological conditions. Peptidomic studies of mouse brain regions from knockout mice lacking *Cpe *(*Cpe*^fat/fat^) reveal lower levels of most secretory pathway peptides, indicating a major role of CPE in peptide biosynthesis (5). In addition to its role in neuronal development, CPE is also neuroprotective (6,7). *Cpe*^fat/fat^ mouse models, with an inactive CPE enzyme due to point mutations in *Cpe*, have obesity, age-dependent infertility, hyperglycaemia, disorders of bone metabolism, inflammatory bowel disease and neurological abnormalities (3,8,9). *CPE* is located on chromosome 4q32.3 and has nine exons. To date, only one mutation, a frameshift homozygous mutation (c.76_98del; p.E26RfsX68) within *CPE*, has been described in a patient with morbid obesity, type 2 diabetes mellitus, intellectual disability and hypogonadotropic hypogonadism; this is not listed as a specific Mendelian syndrome in the Online Mendelian Inheritance in Man (OMIM) database (10).

Clinical and genetic heterogeneity in obesity and intellectual disability represents a major diagnostic challenge. Although the presence of distinct clinical features may help in identifying a specific cause in some cases, most patients remain undiagnosed. Many genes have been linked to underlying Mendelian aetiology; however, the diagnostic power of genome sequencing remains limited, ranging from 8-70% of cases (11). Whole exome sequencing (WES) is a powerful tool identifying many single gene disorders, including genetic causes for patients with syndromic obesity. Here we present WES results of three Turkish siblings, born to consanguineous parents, having obesity, intellectual disability, and hypogonadotropic hypogonadism, revealing a homozygous nonsense mutation in *CPE*. These cases support the previously reported syndromic case with a truncating mutation, which suggests a new monogenic obesity syndrome. Therefore,* CPE* deficiency should be considered in such cases.

## Methods

### Cases

**Figure 1:**
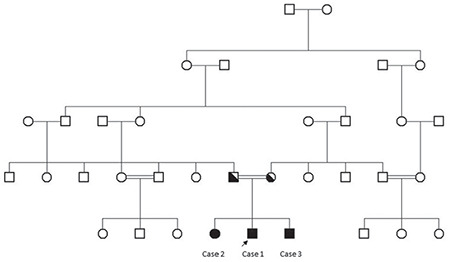
Pedigree of the family

The index case was a 15-year-old boy (Case 1), who has an affected 19-year-old sister (Case 2) and an affected three-year-old brother (Case 3). Consanguineous marriage was confirmed between their parents as shown in the pedigree (Figure 1). Peripheral blood samples were collected from the patient and his siblings for routine haematological and biochemical examination and DNA sequencing analysis. Informed consent was obtained from the parents for genetic analysis and for publishing their photographs in this study.

### Case 1

**Figure 2:**
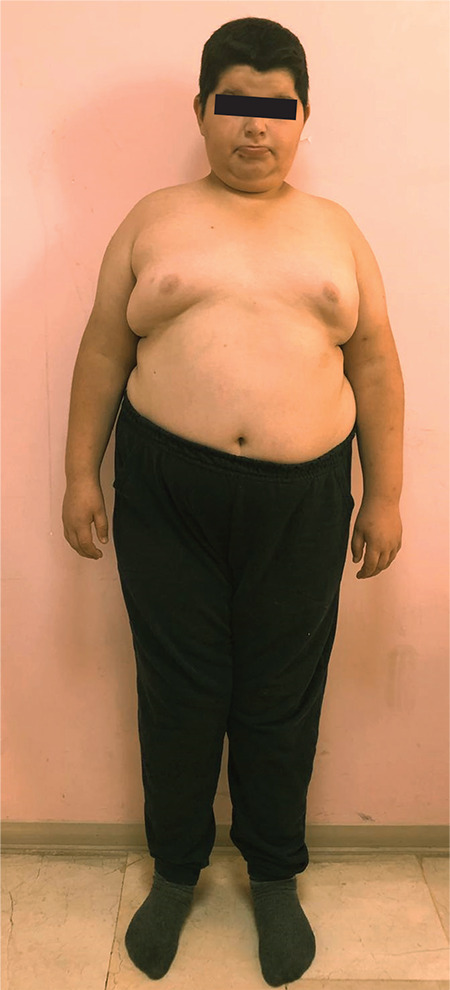
General appearance of the index case

**Figure 3:**
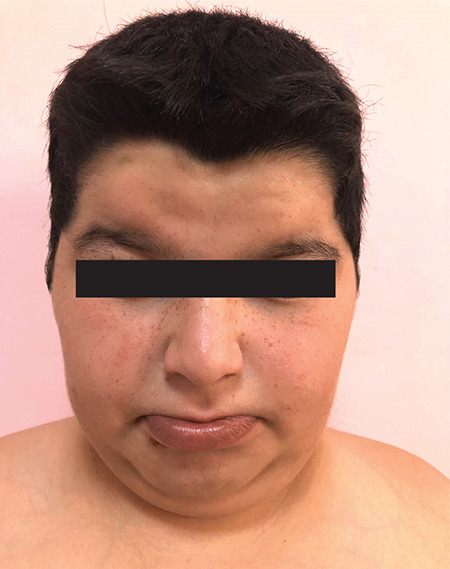
Facial appearance of the index case

The index case was referred to paediatric endocrinology with obesity, hypothyroidism, micropenis, undescended testes and intellectual disability at the age of 11 years. He was the second child from a first cousin marriage. He was born on the 38^th^ week of gestation by caesarean section. At birth, he weighed 3200 g [-0.4 standard deviation score (SDS)] and measured 50 cm in length (0 SDS). Medical history revealed delayed developmental milestones. He had significant hypotonicity at infancy. He achieved head control at 1-year-old and could walk at five-year-old. He had marked speech delay and could say a few words only. Excess weight gain was reported after four years of age due to increased appetite. He used a ventilation tube due to recurrent otitis media. Hypothyroidism was diagnosed at nine years of age. Physical examination showed his weight was 62.8 kg (2.1 SDS), height was 139.5 cm (-0.78 SDS), body mass index (BMI) was 32.2 kg/m^2^ (2.4 SDS) and target height was 170.2 cm (-0.96 SDS) (Figure 2). Dysmorphic examination showed that he had a round face, full cheeks, micrognathia, gynaecomastia and micropenis (Figure 3). His stretched penile length was 3.5 cm (<3^rd^ percentile) with cryptorchidism (suprascrotal gonads) of 1 mL volume. His laboratory findings were as follows: morning fasting glucose 80 mg/dL, insulin 66 uU/mL, HOMA-IR 11.8 (severe insulin resistance), thyroid stimulating hormone (TSH) 0.07 mU/L and free thyroxine (FT4) 0.59 ng/dL, suggesting central hypothyroidism (Table 1). Thyrotropin releasing hormone stimulation test revealed that the TSH response was 0.83, 0.85 and 0.41 mU/L at 30, 60 and 90 min respectively. A diagnosis of central hypothyroidism was made. Upon evaluating other pituitary hormone deficiencies, the following was reported: early morning (08:00 am) prolactin 6.9 ng/mL, follicle-stimulating hormone (FSH) 0.76 mIU/mL, LH 0.22 mIU/mL, total testosterone 0.1 ng/dL, adrenocorticotropic hormone (ACTH) 31.2 pg/mL and cortisol 6.1 µg/dL. Corticotrophin releasing hormone (CRH) stimulation test showed a normal peak cortisol response of 22.1 µg/dL, which excluded central adrenal insufficiency. On his last visit at the age of 15 years, he weighed 96 kg (+2 SDS), was 156 cm (-2 SDS) tall and had a BMI of 39.4 kg/m^2^ (+3.28 SDS). He remained prepubertal and had low levels of gonadotropins with micropenis and cryptorchidism. His initial diagnosis was hypogonadotropic hypogonadism and a luteinising hormone (LH) releasing hormone (LHRH) stimulation test was planned.

Cranial/hypophysial magnetic resonance imaging (MRI), abdominal ultrasonography, skeletal survey, echocardiography and eye examination were normal. Chromosomal analysis revealed a normal male karyotype (46,XY). Differential diagnosis fluorescence in situ hybridization analysis for Prader-Willi syndrome and chromosomal microarray analysis were found to be normal. According to the clinical and laboratory findings of the index case and similar clinical findings in his 19-year-old sister, with intellectual disability and obesity, and three-year-old brother, with global developmental delay and central hypothyroidism, it was thought that a differential diagnosis for obesity and mental retardation syndromes, with an autosomal recessive inheritance pattern, should be established.

### Case 2

**Figure 4:**
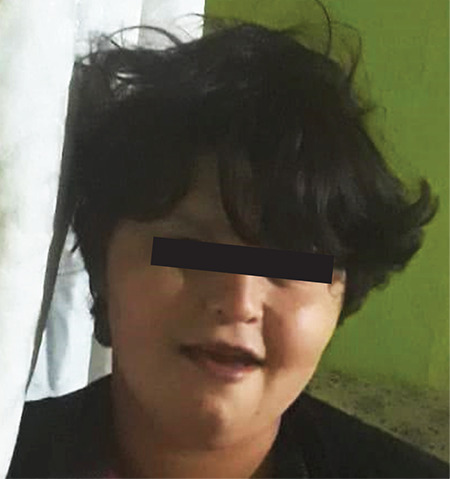
Facial appearance of affected sister (Case 2) of the index case

The proband’s 19-year-old sister was delivered at full term by caesarean section with appropriate birth weight. Her developmental milestones were delayed. She started to walk at six-year-old, used a few words and could not form sentences. Her history revealed puberty tarda, with a primary amenorrhea. Physical examination showed that her weight and height were 78 kg (>97^th^ centile) and 160 cm (25-50^th^ centile), respectively. She was obese with a BMI of 30.4 kg/m^2^. She was Tanner stage 2. She had intellectual disability. Physical examination revealed full cheeks, micrognathia and round face as dysmorphic features (Figure 4). In the most recent laboratory examination, insulin resistance and central hypothyroidism were detected. Her LH value was within normal range, but she had insufficient estrogen values. Whether this is a late onset puberty, or a pause of puberty can’t be differentiated. Further stimulation tests were planned.

### Case 3

**Figure 5:**
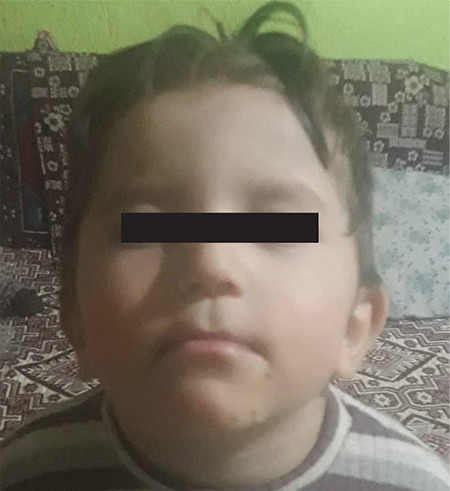
Facial appearance of affected brother (Case 3) of the index case

The proband’s three-year-old brother was born at the 38^th^ week of gestation via caesarean section with a weight of 3200 g. During the neonatal period, he was diagnosed with congenital hypothyroidism on the 15^th^ day of life. He was hypotonic and at two months old he had a history of aspiration. Medical history revealed delayed developmental milestones; he could not walk or talk and had been sitting up for only six months. Physical examination showed that he weighed 18 kg (1.9 SDS), was 99 cm (1.5 SDS) tall and had a BMI of 16.32 kg/m^2^ (0.26 SDS). On his genital examination bilateral testicles were palpable within the scrotum with a volume of 2 mL. Dysmorphic features included round face, full cheeks, large ears and micrognathia (Figure 5). Thyroid function tests were FT4 0.03 ng/dL and TSH 0.92 mU/L, indicating central hypothyroidism. Other laboratory biochemical parameters were normal; FSH 0.83 mIU/mL, LH 0.12 mIU/mL, ACTH 23.2 pg/mL and cortisol 9.6 µg/d. Since he was pre-pubertal, it was impossible to detect whether he had hypogonadotropic hypogonadism. The pituitary MRI was normal. Echocardiography showed atrial septal defect and patent ductus arteriosus.

### Whole Exome Sequencing

Next-generation sequencing was performed for WES analysis using the Ion S5™ Sequencer (Thermo Fisher Scientific, Inc., Wilmington, DE, USA). The Ion AmpliSeq™ Exome RDY kit (Thermo Fisher Scientific, Inc., Wilmington, DE, USA) was used, according to the manufacturer’s protocol. The Ion reporter software v.5.2 (Thermo Fisher Scientific) was used to analyse the mutations (https://ionreporter.thermofisher.com/ir/). Under an assumed autosomal recessive mode of inheritance, all variants were assessed individually according to the clinical phenotype, minor allele frequency (MAF) score and pathogenicity scores were calculated using prediction programmes. Variants were filtered to retain non-synonymous changes with a MAF of <0.01 using combined datasets from the 1000 Genomes Project, the Exome Variant Server project and the Genome Aggregation Database (GnomAD). The potential functional impact of the disease candidate variants were assessed using SIFT (http://sift.jcvi.org/), PolyPhen-2 (http://genetics.bwh.harvard.edu/pph2/), MutationTaster (http://www.mutationtaster.org/) and VarSome (https://varsome.com/). All genetic variants were screened for pathogenicity, mode of inheritance and clinical phenotypes. Finally, candidate pathogenic variants identified by WES were verified with Sanger sequencing.

### Validation by Sanger Sequencing

Sanger sequencing was used to validate the novel *CPE *mutation using the 3130 genetic analyser (Applied Biosystems, Foster City, USA). Primers designed to amplify exon 2 of *CPE* (NM_001873.4) are as follows: forward, 5’-TGTAGGTATACAATATATTTGGCTCTG-3’ and reverse, 5’-CCATCTGTAAGCTTTGTGCG-3’. The sequencing results were analysed using the Genomics Workbench 20.0 (QIAGEN) (https://digitalinsights.qiagen.com/). Sanger sequencing was also performed in the affected brother and sister and the parents to evaluate segregation in the family.

## Results

**Figure 6:**
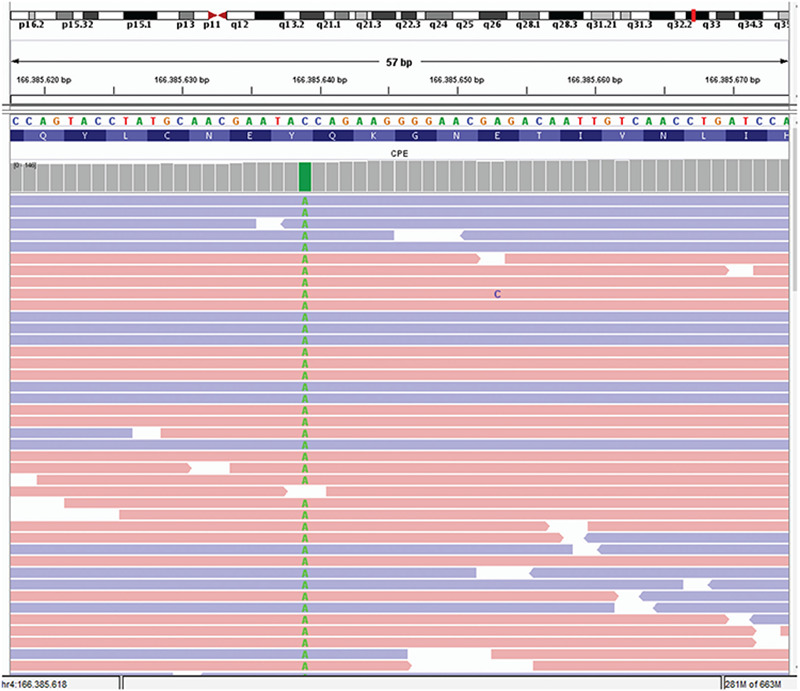
Next generation sequencing analysis of the proband showing homozygous c.405C>A (p.Y135*) mutation in *CPE* gene

**Figure 7:**
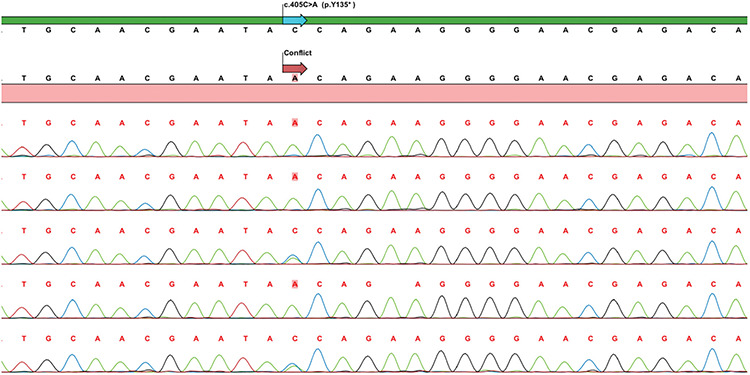
Validation and segregation analysis of the family by Sanger sequencing revealing c.405C>A (p.Y135*) (NM_001873) mutation. (Line 1: index case, Line 2: Case 2, Line 3: Mother, Line 4: Case 3, Line 5: Father)

WES analysis, performed due to a lack of preliminary diagnosis of the index case, revealed 52,465 unfiltered variants initially. An average coverage at >140 × read depth for 96% of the exome was attained. The variants were filtered out according to zygosity, MAF, location and type of mutation. Thereafter, 14 variants in seven genes were found to be potential disease-causing variants and classified as pathogenic or variants of unknown significance, according to the American College of Medical Genetics guidelines (Table 2). Initial analysis revealed 2 homozygous and hemizygous nonsense mutations in the *CPE* and* COL4A6* genes, respectively. After interpretation of these variants with the scope of clinical presentation of the case, homozygous nonsense (NM_001873.4):c.405C>A (p.Y135*) mutation in *CPE *gene (Chr4:166385639-C/A) was considered to be the candidate disease-causing variant (Figure 6). Mutations in *COL4A6 *is associated with deafness which was not found in our patient. The *CPE* variant was not found in the Exome Aggregation Consortium, GnomAD exomes or genomes, and in our 100 in-house controls. The gene was not linked to a specific human disease in the OMIM database. The homozygous nonsense mutation showed high pathogenic scores in prediction software, such as VarSome, Mutation Taster and FATHMM-MKL, suggesting a strong causative role in the pathogenicity of the disease (Table 3) (12,13). After confirming the mutation by Sanger sequencing, the siblings and parents of the index case were also sequenced (Figure 7). The affected siblings were homozygous and the parents were heterozygous for the mutation.

## Discussion

In this study, we present three affected siblings having the same homozygous c.405C>A (p.Y135*) mutation in *CPE*, which can be classified as a distinct syndromic obesity gene. CPE is a neuropeptide-processing enzyme, expressed abundantly in neural and endocrine tissues (14). CPE plays an important role during embryonic and postnatal brain development as a neuroprotective factor. CPE is also known as NF-α1 as it induces ERK and Akt signalling, similar to classic trophic factors, such as BDNF or nerve growth factor (13). CPE was thought to function intracellularly to process a precursor protein with neuroprotective activity besides its role as an extracellular neuroprotective trophic factor, independent of its enzymatic activity (15). A novel role for CPE in the development and branching of proximal dendrites, necessary for cortical neuron migration and dendritogenesis, has been proposed (6). CPE also has functions in prohormone sorting and vesicle transport in the endocrine and nervous system (7,14). The neuroprotective role of *Cpe* has been demonstrated in knockout mouse models showing severe neurodevelopmental delay, neurodegeneration and depression. Cases presented in this study showed severe neurodevelopmental delay that may be linked to a loss of enzymatic activity of CPE and may be linked to defective cortical neuron development.

The *Cpe*^fat/fat ^mice exhibit a decrease in CPE levels and an increase in proinsulin levels, thereby confirming the role of CPE in insulin dysregulation (16,17). A spontaneous point mutation in *Cpe*, which diminishes its enzymatic activity, results in severe obesity; thus, the model carrying this mutation is called the *Cpe*^fat^/*Cpe*^fat^ mouse model (18). In another mouse model,* Cpe *knockout mice, in which exons 4 and 5 are deleted, showed insulin resistant diabetes, obesity, infertility and neurological and behavioural abnormalities (19). The *Cpe*^fat^/*Cpe*^fat^ mouse model exhibits elevated levels of hormones and neuropeptide precursors and decreased levels of bioactive peptides, suggesting that CPE has a role in the processing of prohormones and proneuropeptides (16,17,18,20). *Cpe*^fat/fat^ mice with p.Ser202Pro mutation present with endocrinological abnormalities, such as obesity, diabetes and infertility due to the lack of enzyme activity and display a variety of behavioural abnormalities (21). In another study, a mutation in CPE-NFα1, consisting of three adenosine inserts, introduced nine amino acids, including two glutamines into the mutant protein, called CPE-QQ, and resulted in its accumulation in the endoplasmic reticulum (ER) and the subsequent degradation by proteasomes. Mice having this mutation show neurodegeneration in the hippocampus and prefrontal cortex, deficits in neurogenesis at the dentate gyrus and hyperphosphorylation of tau protein (22). A mutation in human *CPE-NF*α*1*, c.T980C (p.W235R), causes a loss of its enzyme activity and neurotoxic accumulation in the ER, which results in ER stress and cell death and finally, neurological disorders (23).

Only one study has demonstrated a case with a null mutation having similar symptoms to the three siblings, including obesity, diabetes, hypogonadotropic hypogonadism and impaired intellectual ability (10). Together with the previous case, we may conclude that the nonsense mutation detected in our index case resulted in a nonsense-mediated mRNA decay and the complete absence of a functional CPE protein, which caused the syndromic features of the cases. Polymorphism studies revealed an association of *CPE* variants with BMI or non-insulin dependent type 2 diabetes mellitus in different cohorts (24,25). A missense c.847C>T (p.Arg283Trp) variant was detected in type 2 diabetes mellitus in Ashkenazi families, which was linked to hyperproinsulinaemia and diabetes (26). Both *Cpe* knock out mice models and polymorphism studies in humans support the role of CPE in endocrinological defects, such as obesity, hypothyroidism and hypogonadotropic hypogonadism, which are present in our cases.

*CPE* is not linked to a specific Mendelian syndrome in the OMIM database. During WES data interpretation, if only OMIM genes were analysed by analysing software programs, this variant may have been filtered out. It is necessary to search the databases to support a correlation between potential disease-causing variants and clinical findings in the index case. In our case, as this variant has a high score in the ACMG guidelines variant classification, we focused on this variant by validating in the index case and segregation in the family. The variant found in our family may improve the diagnostic yield in patients with this syndromic obesity.

### Study Limitations

Our results should be supported with more cases with variations in *CPE* and obesity, intellectual disability and hypogonadotropic hypogonadism. Moreover, functional studies should be performed using *in vivo* models.

## Conclusion

Obesity and intellectual disability have clinical and genetic heterogeneity. In this report we present three siblings having obesity, intellectual disability and hypogonadotropic hypogonadism with a novel mutation in *CPE*, which is not linked to a specific human Mendelian disease. Together with the findings from a previous case, *CPE *can be considered as a candidate gene for a new monogenic obesity syndrome.

## Figures and Tables

**Table 1:**
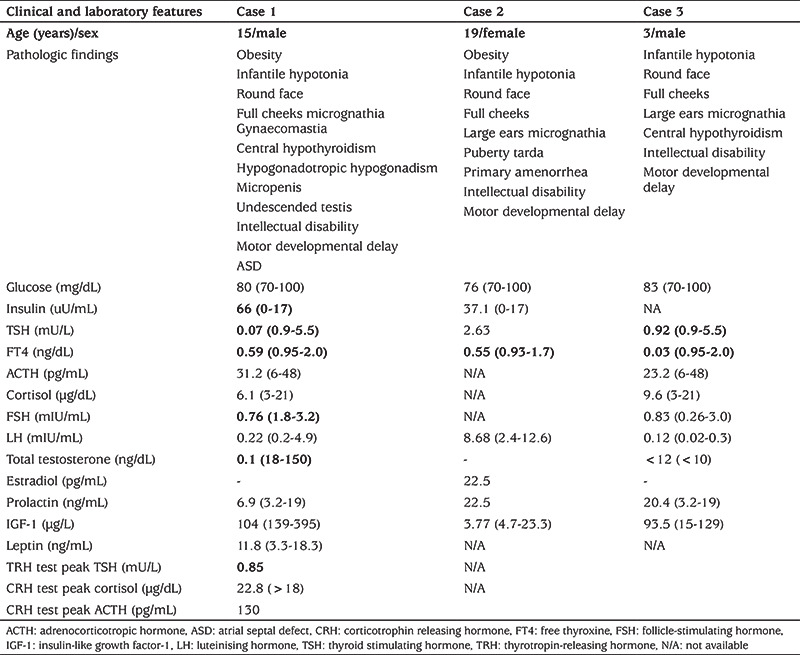
Clinical and laboratory findings of patients

**Table 2:**
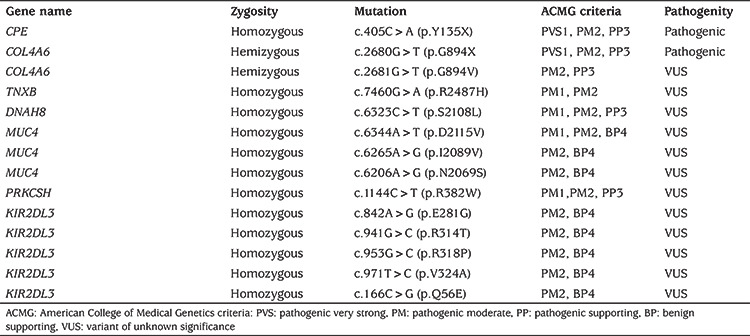
List of pathogenic variants after filtration strategy

**Table 3:**
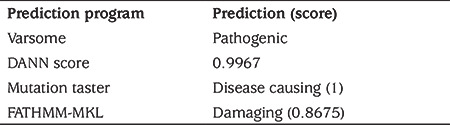
Results of prediction programs for the CPE mutation c.405C>A (p.Y135X)
